# Development of vaccine-induced immunity against TRT in turkeys depends remarkably on the level of maternal antibodies and the age of birds on the day of vaccination

**DOI:** 10.1186/s12917-015-0345-5

**Published:** 2015-02-07

**Authors:** Marcin Smialek, Daria Pestka, Bartlomiej Tykalowski, Tomasz Stenzel, Andrzej Koncicki

**Affiliations:** Department of Poultry Diseases, Faculty of Veterinary Medicine, University of Warmia and Mazury, Oczapowskiego 13/14, 10-719 Olsztyn, Poland

**Keywords:** Avian metapneumovirus, Turkeys, Vaccination, Humoral immunity, Cell mediated immunity, Maternally derived antibodies

## Abstract

**Background:**

Avian Metapneumovirus (aMPV) infections are a huge economical issue for the poultry industry worldwide. Although maternal antibodies do not protect turkey poults against turkey rhinotracheitis (TRT), almost no studies have been conducted so far regarding the impact of these antibodies on vaccine induced immunity development against aMPV infection. We conducted four experiments on commercial turkeys aimed at comparing local humoral and cell mediated immune response of maternally delivered anti-aMPV antibody positive (MDA+; Experiment I and II) and negative (MDA-; Experiment III and IV) turkeys following vaccination with an attenuated live aMPV subtype A vaccine at the day of hatch (Experiment I and III) or at two weeks of age (Experiment II and IV).

**Results:**

Regardless of the birds’ age, vaccination of MDA- turkeys resulted in strong stimulation of CD8^+^ T lymphocytes in the Harderian gland and tracheal mucosa, whereas vaccination of MDA+ birds stimulated mainly CD4^+^ T cells in those structures. An increase in the level of anti-aMPV IgY antibodies was noted in the serum (but not in tracheal washings) as early as 7 days after vaccination, but only in birds possessing low levels (MDA+ birds vaccinated at 2 weeks of age) or no maternal anti-aMPV antibodies at the time of vaccination. In MDA+ turkeys vaccinated at hatch, the decrease in serum levels of maternal anti-aMPV antibodies proceeded faster (in comparison to control group), which, together with faster viral clearance, indicates that maternal antibodies can inhibit vaccine virus replication and influence the development of vaccine-induced immunity.

**Conclusion:**

This study provides the first documented evidence that the frequency of TRT outbreaks in the field and/or failure of TRT vaccination could be correlated with differences in the immunological status and/or age of vaccinated turkeys.

## Background

The avian metapneumovirus (aMPV), a member of the family *Paramyxoviridae*, genus *Metapneumovirus* [1], is a strongly infectious RNA virus that causes turkey rhinotracheitis (TRT) in flocks of turkeys and the swollen head syndrome (SHS) in chickens. aMPV infections cause massive financial losses in the poultry industry worldwide. aMPV was first identified in South Africa in 1978 [2], and since then, it has spread to numerous countries, excluding Australia and Canada. aMPV has been classified into 4 subtypes (A–D) based on its nucleotide sequence and antigen structure [3-5].

In most immunoprophylaxis programs, one-day-old turkey poults are administered live attenuated vaccines against TRT by coarse spray to provide the earliest possible protection of the upper respiratory tract against aMPV infections [6]. Despite the above, TRT outbreaks in the field are noted very frequently [7-9].

Humoral immunity is strongly stimulated by vaccination or aMPV infection [7,10-13], but antibodies do not play a key role in protection against TRT and should not be considered as indicators of immunity against aMPV infections [7,9,11,12,14-16]. It has been shown, however, that high antibody titers suppress aMPV replication in the upper respiratory tract, thus alleviating the clinical course of TRT [6,11,12,14]. In one-day-old turkey poults, most of which originate from parent flocks vaccinated against TRT, the possible influence of maternal antibodies on the development of vaccine-induced immunity against aMPV is questionable.

Cell-mediated immunity (CMI) is increasingly often considered as the decisive factor in protection against TRT. Unfortunately, little is known about local CMI mechanisms in turkeys’ upper respiratory tract during infections and after vaccination against TRT. These mechanisms seem to play a particularly important role because aMPV infects the host through mucosal sites in the upper respiratory tract. Liman and Rautenschlein [7] demonstrated a significant increase in the percentage of CD4^+^ T lymphocytes with concurrent upregulation of IL-6 and/or IFN-γ in the Harderian gland (HG) after vaccination or infection with aMPV/A or B. Conversely, Cha [17] reported an increase in the percentage of CD8^+^ T cells, but not in CD4^+^ T cells, in the upper respiratory tract after aMPV/C inoculation. The cited results indicate that CMI actively participates in protection against aMPV infections and that local CMI mechanisms may be related to age and the aMPV subtype.

In view of the diverse immunopathogenesis of TRT, the aim of this study was to describe selected parameters of local CMI and humoral immunity in the upper respiratory tract of turkeys immunized against TRT with live attenuated aMPV/A vaccines. The specific goal of the study was to characterize the development of vaccine-induced immunity in variously-aged birds and in turkeys with, or without maternal anti – aMPV antibodies on the day of vaccination.

## Methods

The experimental procedures and animal handling procedures were conducted with the approval of the Local Ethic Committee for Animal Experiments in Olsztyn, Poland (resolution No. 37/2011).

### Turkeys and vaccination

The experiments were carried out on 568 commercial Hybrid Converter turkeys of both sexes. The birds were not vaccinated at hatchery, and the absence of aMPV genetic material in samples collected at hatch was confirmed by nested RT-PCR. The MDA+ group comprised 284 turkeys that were positive for maternally derived antibodies and were obtained from a parent flock vaccinated against TRT (3 times with live aMPV/A vaccine and twice with inactivated vaccine). MDA+ birds were provided by the Grelavi S.A. hatchery (a Hendrix Genetics Company) in Kętrzyn, Poland. The MDA- group consisted of 284 turkeys that were negative for maternally derived antibodies and originated from a parent flock not vaccinated against TRT (reproductive flock from Canada). MDA- birds were provided by the Gerczak Nord-Pol Hatchery in Laseczno, Poland.

Turkeys were housed in isolated units maintained at a biosafety PCL 3 facility. Water and feed were given to birds *ad libitum*. Birds were vaccinated with aMPV/A strain BUT1 #8544 of lyophilized attenuated commercial vaccine. Each bird was inoculated with 10^4^ of tissue culture infectious doses (TCID_50_) per bird oculonasally. Unvaccinated birds received sterile vaccine diluent.

### Experimental design

#### Experiment I

Experiment I was carried out on 160 one-day-old MDA+ turkeys. The birds were randomly divided into vaccinated (MDA+0/V) and unvaccinated (MDA+0/NV) groups. Before vaccination, samples were collected from 24 randomly selected turkeys for further analysis. The day of vaccination (hatch day) was termed zero day of life (DOL), and the results for samples collected on this day were marked as zero day post-vaccination (DPV).

Birds of both groups were raised to 14 DOL, and further samples were collected on 3, 7 and 14 DPV (from 20–24 birds in each group in each sampling).

In each sampling, choanal swabs (e-Swab, Copan Diagnostics, Canada) were collected from 5 birds for RT-PCR analysis (swabs were stored at −76°C until further analysis) and blood samples were collected from 15 birds. The birds were euthanized, and HG and trachea (TC, cut from the larynx to the cranial aperture of body cavity) were collected. HG and TC were pooled from 4–6 birds per sample (the number of birds per sample were equal in each sampling), which produced 4 samples of each organ for flow cytometry analysis. Tracheal washings were collected from 10 birds in each group by passing 1 ml of phosphate-buffered saline (PBS, pH 7.2, Sigma Aldrich, Germany) through the lumen of the trachea with a 2 ml syringe. The trachea was massaged gently to intensify the washing effect. Following centrifugation, the supernatant and serum samples were stored at −20°C for further use.

#### Experiment II

The experimental design was identical to that of Experiment I. The experiment was carried out on 124 MDA+ turkeys raised until 14 dol. On 14 DOL, samples were collected from 20 birds, and the remaining turkeys were divided randomly into vaccinated (MDA+14/V) and unvaccinated (MDA+14/NV) groups. Similarly to Experiment I, the results reported for samples that were collected before vaccination (14 DOL) represent 0 DPV, and further samples were collected on 3 (17 DOL), 7 (21 DOL) and 14 (28 DOL) DPV (from 16–20 birds in each group in each sampling).

#### Experiment III

Experiment III was carried out on 160 one-day-old MDA- turkeys. The experimental design and schedule, the day of vaccination and the sample collection protocol were identical to those described in Experiment I. Turkeys were divided into two groups of vaccinated (MDA-0/V) and unvaccinated (MDA-0/NV) birds.

#### Experiment IV

Experiment IV was carried out on 124 fourteen-day-old MDA- turkeys. The experimental design and schedule, the day of vaccination and the sample collection protocol were identical to those described in Experiment II. Turkeys were divided into two groups of vaccinated (MDA-14/V) and unvaccinated (MDA-14/NV) birds.

### Isolation and determination of mononuclear cell counts

HG were homogenized in a manual Dounce tissue grinder in 3 ml of complete cell culture medium (RPMI – 1640, 10% Fetal Bovine Serum (FBS), 1% MEM Non-essential Amino Acids solution, 1% Penicillin – Streptomycin, 1% HEPES, 1% Sodium pyruvate, Sigma Aldrich, Germany) and filtered (70 μm mesh). After centrifugation in culture medium at 450 g for 10 min at 20°C, the supernatant was discarded and cell pellets were resuspended in 3 ml of 40% Percoll density gradient and gently layered on 3 ml of 60% Percoll. Percoll concentrations were obtained by combining 100% Percoll solution (Percoll with 10% Hank’s balanced salt solution, Sigma Aldrich, Germany) with the appropriate amount of culture medium. Mononuclear cells were collected from the interphase after density centrifugation (20 min, 1900 g, 20°C) and washed twice in PBS with 5% FBS.

TC were cut into 2–3 mm pieces and placed in 100 ml an Erlenmeyer flask with 25 ml of DL-Dithiothreitol solution (RPMI – 1640, 5% FBS, 1% HEPES, 2 mM DL-Dithiothreitol, Sigma Aldrich, Germany) and incubated at room temperature for 5 min to remove epithelial cells with intraepithelial lymphocytes. TC were filtered (70 μm mesh), and flasks were rinsed with RPMI – 1640 before collagenase treatment. TC were placed in 25 ml of Collagenase type IV solution (RPMI – 1640, 1% HEPES, 1% Penicillin – Streptomycin, 200 Collagen Digestion Units/ml, Sigma Aldrich, Germany) and incubated at 38°C for 35 min to isolate lamina propria lymphocytes. TC were filtered (70 μm mesh) after incubation. Filtrates from both incubations were washed three times in culture medium. Final pellets of intraepithelial lymphocytes were merged with lamina propria lymphocytes from the same sample, they were washed and resuspended in 3 ml of 40% Percoll. The isolation procedure was identical to that involving HG. The obtained HG and TC mononuclear cells were resuspended in 1 ml of PBS, and the absolute lymphocyte counts (ALC)/ml were calculated for each sample with the Vi-cell XR (Beckman Coulter, USA) cell counter and cell viability analyzer.

### Flow cytometry and dual platform analysis

2.5 × 10^5^ (or 1.25 × 10^5^, depending on the ALC) of viable mononuclear cells from HG or TC were stained with monoclonal Mouse anti-Chicken CD4 - FITC (clone 2–35) and CD8 - PE (clone 11–39) for T lymphocytes, or polyclonal Goat anti-Chicken IgM - FITC for B cells (AbD Serotec, UK), incubated for 30 min on ice and washed twice in PBS. The cells were analyzed with the FACSCanto II (BD, USA) flow cytometer.

Data acquisition was performed in FACSDiva Software 6.1.3. (BD, USA). Cells were analyzed and immunophenotyped in the FloJo 7.5.5 (Tree Star, USA). Relative cell counts (RCC) of T and B lymphocytes in HG and TC were established in the above environment.

Absolute cell counts (ACC) of T and B cells were calculated by dual platform analysis with the use of the following formula: ACC = (ALC * RCC)/100%. Data were expressed as the mean ACC of CD4^+^ and CD8^+^ T lymphocytes or the mean x-fold change in ACC of IgM^+^ B lymphocytes in vaccinated groups relative to control groups.

### Enzyme-linked immunosorbent assay (ELISA)

Sera and tracheal washings were tested for specific IgY anti-aMPV antibodies with the APV Ab ELISA kit (IDEXX Laboratories, USA). Sera of MDA- birds were diluted 100-fold rather than 500-fold (manufacturer’s recommendations) to enhance detection of low antibody levels. 100-fold dilution, as a comparative to 500-fold, was also used in analyses of MDA+14/V and NV serum. Tracheal washings were incubated undiluted. Successive steps of the procedure were performed according to the manufacturer’s recommendations. ELISA was carried out with the use of the Eppendorf epMotion 5075 LH automated pipetting station (Eppendorf, Germany), BioTek ELx405 automatic plate washer (BioTek, USA) and BioTek ELx800 plate reader. The sample to positive (S/P)-ratio was calculated based on the ODs and used to express the mean (S/P)-ratio +/− SD per group and per DPV.

### Nested RT-PCR

Nested RT-PCR was performed in accordance with the protocol described in a previous study [18] with minor modifications. RNA was extracted from choanal swabs with the RNeasy Mini Kit (Qiagen, Germany) according to the manufacturer’s recommendations. The concentrations and purity of extracted RNA were evaluated with the NanoDrop 2000 spectrophotometer (ThermoScientific, USA). Samples ranging from 1.8 to 2.0 of the OD 260/280 ratio were used for further analysis. RT was performed with the EnhancedAvian HS RT-PCR Kit (Sigma-Aldrich, USA) according to the manufacturer’s recommendations. PCR was performed with the use of the HotStarTaq Plus Master Mix (Qiagen, Germany) and the vapo.protect (Eppendorf, Germany) thermocycler. The first PCR was carried out with 10 μl of HotStarTaq *Plus* DNA, 0.1 μl of 100 μM primers G1+A and G6, 6.8 μl of RNase-free water and 3 μl of cDNA. Nested PCR was performed with 10 μl of HotStarTaq *Plus* DNA, 0.1 μl of 100 μM primers G8+A and G5, 2 μl of CoralLoad PCR, 5.8 μl RNase-free water and 2 μl of the amplicon from the first PCR. Primer sequences and product sizes for every PCR are summarized in Table 1. Both PCRs had the following thermal profile: 95°C for 5 min, 30 cycles of 94°C for 1 min, 54°C for 45 s, 72°C for 45 s, and final elongation at 72°C for 10 min.

**Table 1:** **Sequences of the oligonucleotide primers used in nested RT-PCR analysis and expected PCR product size**

**Primer**	**Sequence**	**Product size**
G1+A	5′-GGGACAAGTATCTCTATG-3′	444 bp
G6	5′-CTGACAAATTGGTCCTGATT-3′	
G8+A	5′-CACTCACTGTTAGCGTCATA-3′	268 bp
G5	5′-CAAAGArCCAATAAGCCCA -3′	

Nested PCR amplicons were analyzed in 2% agarose gel with 0.5 μl/ml of ethidium bromide dye (Sigma-Aldrich, USA). Electrophoresis results were visualized with GelDoc XR+ (Bio-Rad, USA).

### Statistical analysis

The flow cytometry results were processed by Student’s t-test for independent samples in Graphpad Prism 6 software (San Diego, USA) and results of serological examination by non-parametric Mann–Whitney U-test in Statistica PL V10. Differences were considered statistically significant at p ≤ 0.05 and highly significant at p ≤ 0.01.

## Results

### Absolute lymphocyte count

No statistical differences in absolute lymphocyte counts in HG and TC were observed between vaccinated and unvaccinated birds in all experiments, excluding ALC in TC in the MDA-14/V group. In this group, ALC in TC on 14 DPV increased significantly in comparison with control group (data not shown).

### Flow cytometry analysis of T cells

**Figure 1:**
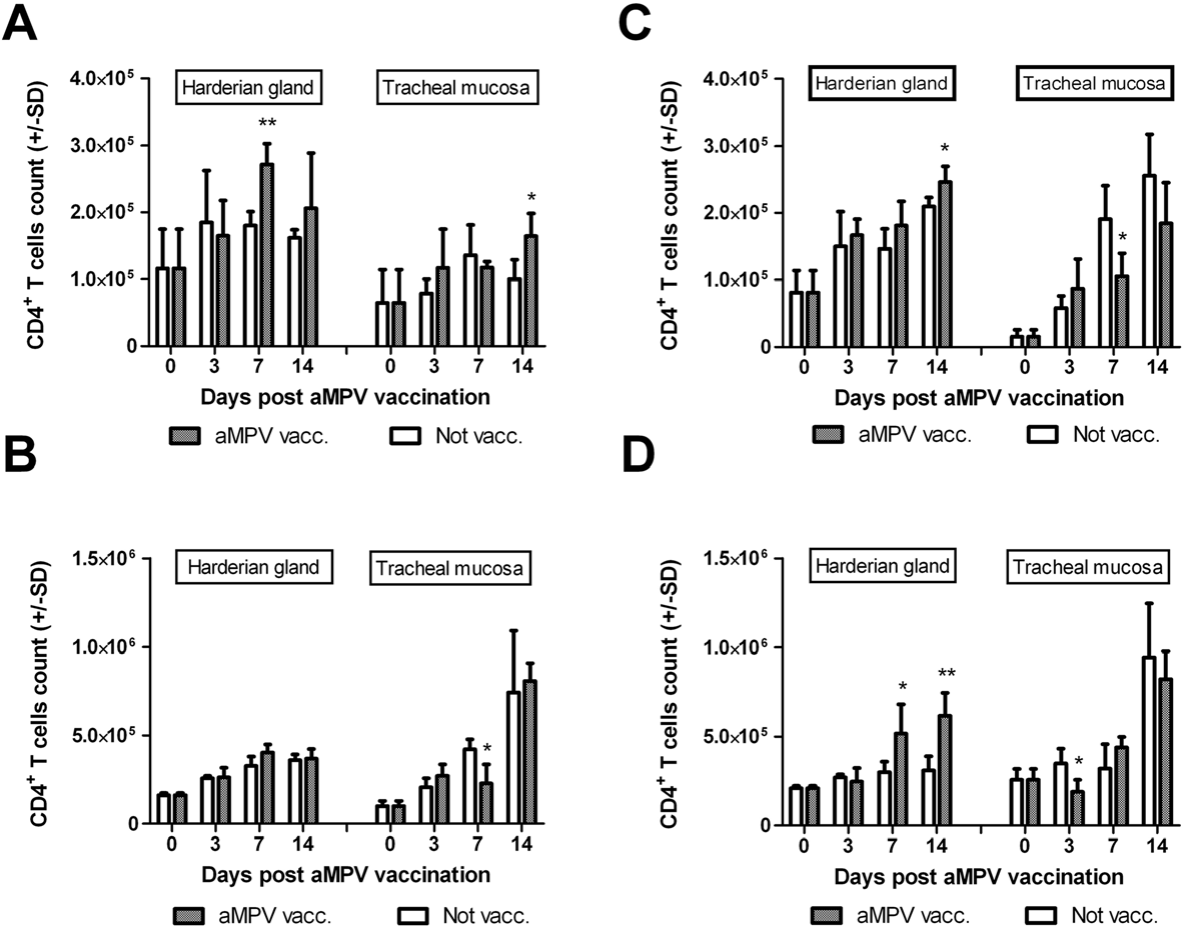
**Summary of CD4** ^**+**^ **ACC in HG and TC (n = 4) at different days post aMPV/A vaccination.** Results for vaccinated (aMPV vacc.) and not vaccinated (Not vacc.) groups in different experiments **(A)** Experiment I; groups MDA+0/V and MDA+0/NV; **(B)** Experiment II; groups MDA+14/V and MDA+14/NV; **(C)** Experiment III; groups MDA-0/V and MDA-0/NV **(D)** Experiment IV; groups MDA-14/V and MDA-14/NV. Results for every experiment are presented as mean CD4^+^ ACC ± SD. */**Significant differences at different DPV (*T*-test, *as p < 0,05, **as p < 0,01).

In Experiment I, a highly significant and significant increase in CD4^+^ T ACC was noted in the MDA+0/V group on day 7 and 14 PV in HG and TC, respectively, in comparison with virus-free birds (Figure 1A). In Experiment II, CD4^+^ T ACC did not increase significantly in vaccinated birds, but a brief increase of this parameter was reported in HG on 7 DPV in the MDA+14/V group. A significant decrease in CD4^+^ T ACC was observed in the MDA+14/V group on 7 DPV in TC in comparison with control (Figure 1B). The noted decrease was short-lived and no significant differences in the value of analyzed parameter were reported between groups in Experiment II on day 14 PV.

In Experiment III, significant increase in CD4^+^ T ACC was noted on 14 DPV in HG in the MDA-0/V group, whereas a significant decrease in this parameter was observed in TC on 7 DPV in comparison with unvaccinated birds (Figure 1C). In Experiment IV, a statistical increase in CD4^+^ T ACC on day 7 PV and a highly significant increase on day 14 PV were reported in HG of MDA-14/V birds, whereas a significant decrease in CD4^+^ T ACC in TC was observed on day 3 PV in comparison with unvaccinated turkeys (Figure 1D).

**Figure 2:**
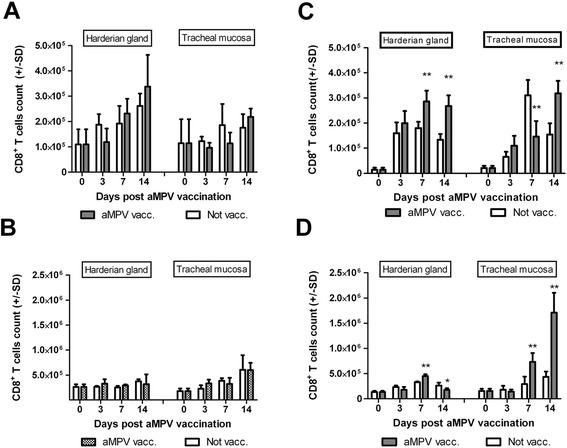
**Summary of CD8** ^**+**^ **ACC in HG and TC (n = 4) at different days post aMPV/A vaccination.** Results for vaccinated (aMPV vacc.) and not vaccinated (Not vacc.) groups in different experiments **(A)** Experiment I; groups MDA+0/V and MDA+0/NV; **(B)** Experiment II; groups MDA+14/V and MDA+14/NV; **(C)** Experiment III; groups MDA-0/V and MDA-0/NV **(D)** Experiment IV; groups MDA-14/V and MDA-14/NV. Results for every experiment are presented as mean CD8^+^ ACC ± SD. */**Significant differences at different DPV (*T*-test, *as p < 0,05, **as p < 0,01).

In Experiments I and II, no significant differences in T CD8^+^ ACC were observed in neither HG nor TC (Figures 2A and B). The number of these cells increased briefly in vaccinated birds (in particular in HG), but the observed changes were not statistically significant.

In Experiment III, a highly significant increase in CD8^+^ T ACC was reported in HG of MDA-0/V birds on days 7 and 14 PV, and a highly significant decrease in the analyzed parameter was observed in TC on 7 DPV in comparison with unvaccinated birds (Figure 2C). The recorded decrease in CD8^+^ T cell count was short-lived, and on 14 DPV, a highly significant increase in this parameter was reported in TC of vaccinated turkeys in comparison with virus-free birds.

The most prominent infiltration of CD8^+^ T cells induced by the vaccine virus was observed in Experiment IV. In the MDA-14/V group, a highly significant increase in CD8^+^ T ACC was noted in TC on days 7 and 14 PV and in HG on 7 DPV in comparison with control. T CD8^+^ cell counts in HG decreased significantly on 14 DPV (Figure 2D).

In Experiments III and IV, significant differences were also observed in double-positive CD4^+^CD8^+^ T ACC (data not shown). In Experiment III, a significant decrease in the absolute counts of double-positive T cells was reported in the MDA-0/V group in TC on day 7 PV. In Experiment IV, a highly significant increase in double-positive T ACC was noted in both HG and TC on 14 DPV. No statistical differences in CD4^+^CD8^+^ T ACC were observed between MDA+ vaccinated groups and unvaccinated birds (data not shown).

### Flow cytometry analysis of B cells

In Experiment I, a significant decrease in B IgM^+^ ACC in TC was observed in MDA+0/V on 7 DPV in comparison with the unvaccinated group (Table 2). The above decrease was transient, and no differences in B IgM^+^ ACC were observed on day 14 PV.

**Table 2:** **Mean** ***x-*** **fold change of IgM** ^**+**^ **B ACC at days post aMPV vaccination** ^**a**^ **in HG and TC**

			**Mean** ***x-*** **fold change** ^**b**^			
**Experiment**	**Group**		**0**	**3**	**7**	**14**
I	MDA+0/V	HG	1	0,72	1,33	0,88
		TC	1	0,83	0,75*	0,88
II	MDA+14/V	HG	1	1,23	1,49*	0,76
		TC	1	1,28	1,32	1,08
III	MDA-0/V	HG	1	1,24	0,85	0,89
		TC	1	1,31	0,91	1,66**
IV	MDA-14/V	HG	1	1,18	0,99	0,74*
		TC	1	0,78	1,17	3,31**

^a^In all experiments vaccinated birds were inoculated oculonassaly with 10^4^ TCID_50_ of live attenuated aMPV/A vaccine.

^b^ *x-*fold change = mean IgM^+^ B ACC of vaccinated group divided by mean IgM^+^B ACC of not vaccinated group.

*/**Significant difference in mean IgM^+^ B ACC of vaccinated birds in comparison to the not vaccinated group (T-test, *as p < 0,05 and **as p < 0,01).

In Experiment II, B IgM^+^ ACC increased significantly in HG of MDA+14/V birds on 7 DPV (Table 2).

In Experiments III and IV, vaccination against TRT induced a highly significant increase in B IgM^+^ cell counts in TC on 14 DPV (Table 2). In Experiment IV, the vaccine virus also caused a significant decrease in IgM^+^ B cell count in HG of MDA-14/V birds on 14 DPV in comparison with control.

### Serology

No statistical differences in the level of specific anti – aMPV antibodies in tracheal washes were observed between vaccinated and not vaccinated birds in any of the experiments (Table 3). In all MDA+ groups on the day of vaccination, in tracheal washes turkeys had detectable maternally derived antibodies (Table 3), and their level decreased with age in large part proportionally to their level in the serum. Only in MDA-14/V group the level of these antibodies increased in tracheal washes on day 14 PV, but this increase was not statistically significant (Table 3).

**Table 3:** **Tracheal washings anti – aMPV IgY antibody level after aMPV vaccination** ^**a**^ **of turkeys**

		**Mean (S/P)-ratio ± S.D.** ^**b**^ **at days post aMPV/A vaccination**			
**Experiment**	**Group**	**0**	**3**	**7**	**14**
I	MDA+0/V	1.152 ± 0.995	0.446 ± 0.476	0.117 ± 0.136	0.097 ± 0.102
	MDA+0/NV	1.152 ± 0.995	0.598 ± 0.695	0.123 ± 0.204	0.128 ± 0.097
III	MDA-0/V	0.00 ± 0.00	0.00 ± 0.00	0.00 ± 0.00	0.005 ± 0.011
	MDA-0/NV	0.00 ± 0.00	0.00 ± 0.00	0.00 ± 0.00	0.00 ± 0.00
II	MDA+14/V	0.128 ± 0.097	0.025 ± 0.036	0.022 ± 0.033	0.023 ± 0.025
	MDA+14/NV	0.128 ± 0.097	0.025 ± 0.039	0.031 ± 0.039	0.025 ± 0.042
IV	MDA-14/V	0.00 ± 0.00	0.00 ± 0.00	0.00 ± 0.00	0.029 ± 0.058
	MDA-14/NV	0.00 ± 0.00	0.00 ± 0.00	0.00 ± 0.00	0.00 ± 0.00

^a^In all experiments vaccinated birds were inoculated oculonassaly with 10^4^ TCID_50_ of live attenuated aMPV/A vaccine.

^b^10 samples of undiluted tracheal washings per group were analyzed.

In Experiment I, high level of anti-aMPV antibodies were reported in the serum and tracheal washes on the day of vaccination. In the MDA+0/V group, mean (S/P)-ratios in the serum and tracheal washes decreased faster than in unvaccinated birds. In most of cases, the observed decrease was not statistically significant, except on day 3 PV when mean serum antibody level were significantly lower in vaccinated turkeys than in unvaccinated birds (Table 4).

**Table 4:** **Serum anti – aMPV IgY antibody level after aMPV vaccination** ^**a**^ **of turkeys**

		**Mean S/P-ratio ± S.D.** ^**b**^ **at days post aMPV/A vaccination**			
**Experiment**	**Group**	**0**	**3**	**7**	**14**
I	MDA+0/V (1:500)^c^	2.19 ± 1.87	0.9 ± 0.51*	0.57 ± 0.5	0.23 ± 0.16
	MDA+0/NV (1:500)	2.19 ± 1.87	1.9 ± 1.36	0.6 ± 0.62	0.41 ± 0.36
III	MDA-0/V (1:100)	0.001 ± 0.00	0.006 ± 0.01	0.015 ± 0.017	0.179 ± 0.164**
	MDA-0/NV (1:100)	0.001 ± 0.00	0.005 ± 0.005	0.005 ± 0.008	0.00 ± 0.00
II	MDA+14/V (1:100)	0.598 ± 0.492	0.42 ± 0.361	0.21 ± 0.200	0.307 ± 0.217*
	MDA+14/NV (1:100)	0.598 ± 0.492	0.423 ± 0.345	0.228 ± 0.242	0.140 ± 0.116
IV	MDA-14/V (1:100)	0.00 ± 0.00	0.014 ± 0.038	0.034 ± 0.055*	0.123 ± 0.194*
	MDA-14/NV (1:100)	0.00 ± 0.00	0.00 ± 0.00	0.00 ± 0.00	0.00 ± 0.00

^a^In all experiments vaccinated birds were inoculated oculonassaly with 10^4^ TCID_50_ of live attenuated aMPV/A vaccine.

^b^15 samples of serum were analyzed per group.

^c^Sample dilution for ELISA procedure.

*/**Significant difference in mean (S/P)-ratio of vaccinated birds in comparison to the not vaccinated group (U-test, *as p < 0,05 and **as p < 0,01).

In Experiment II, MDA levels were detectable on vaccination day with the mean (S/P)-ratio (at 500-fold dilution) of 0.41 (data not shown), which is twice the recommended (S/P)-ratio (0.2) cut-off value. In the MDA+14/V group, MDA levels increased significantly on 14 DPV in comparison with unvaccinated birds, but differences were noted only when serum were diluted 100-fold (Table 4), and not 500-fold (data not shown).

In Experiment III and IV, anti-aMPV antibodies were not detected in the serum (at 100-fold dilution) or in tracheal washes on the day of vaccination (Tables 3 and 4). Serum level of anti-aMPV IgY antibodies in MDA-0/V increased gradually after vaccination and peaked significantly on 14 DPV (Table 4). Similar results were observed in Experiment IV, where serum antibody levels in MDA-14/V turkeys began to increase significantly (at 100-fold serum dilution) on 7 DPV, and increased further on 14 DPV (Table 4).

### Molecular biology

The effectiveness of aMPV-A vaccine virus detection varied considerably between experiments. Between day 3 and 7 PV in Experiments I and III, aMPV RNA was detected by nested RT-PCR in 100% (5 out of 5) of choanal swabs in both MDA+0/V and MDA-0/V groups. On 14 DPV, aMPV RNA was detected in 40% (2/5) and 100% (5/5) of samples from groups MDA+0/V and MDA-0/V, respectively (Table 5).

**Table 5:** **Detection of aMPV genome in choanal swabs of aMPV vaccinated turkeys by nested RT-PCR**

		**Number of aMPV/A positive birds/total** ^**a**^ **at days post vaccination**			
**Group**	**Experiment**	**0**	**3**	**7**	**14**
MDA+0/V	I	0/5	5/5	5/5	2/5
MDA-0/V	III	0/5	5/5	5/5	5/5
MDA+14/V	II	0/5	2/5	0/5	0/5
MDA-14/V	IV	0/5	0/5	0/5	0/5

^a^In all experiments vaccinated birds were inoculated oculonassaly with 10^4^ TCID_50_ of live attenuated aMPV/A vaccine.

In Experiment II, aMPV RNA was barely detectable in the MDA+14/V group. In this group, genetic material of the vaccine virus was identified only on day 3 PV in 40% (2/5) of choanal swabs. aMPV/A RNA was undetectable on days 7 and 14 PV (Table 5).

In Experiment IV, no viral RNA was detected at any stage of the experiment in choanal swabs of MDA-14/V birds (Table 5). The viral genome was not identified in unvaccinated birds in any experiments (data not shown).

## Discussion

Different TRT vaccination schedules in parent flocks of turkeys are likely to cause highly variable transfer of specific anti-aMPV maternal antibodies to hatching poults. Although these antibodies do not protect turkey poults against TRT, their impact on the development of vaccine-induced immunity against aMPV infections has been investigated by very few studies. The present situation is problematic because most breeder turkey flocks are kept in areas with endemic prevalence of aMPV [19].

Cook et al. [11,12] did not report any differences in the development of post-vaccination immunity between MDA+ and MDA- turkeys immunized with an attenuated aMPV strain (3B (Att.)) on 1 or 7 DOL. However, the attenuated aMPV strain used by the cited authors was characterized by a relatively high virulence index indicated by post-vaccination clinical scoring system. Contemporary TRT vaccines are less pathogenic, and they do not cause such acute side effects [20,21]. Re-evaluating research aiming to compare the development of vaccine-induced immunity to aMPV infections in MDA+ and MDA- birds has not been undertaken to date.

Liman and Rautenschlein [7] demonstrated that the percentage of CD4^+^ T cells subpopulation (but not CD8^+^ T cells) increased briefly in HG of turkeys 7–14 DPV after vaccination with aMPV/B or after infection with aMPV/A or B, which corroborates our results for MDA+ vaccinated birds. The cited authors suggested that the short-lived protection against TRT offered by vaccination and the frequency of TRT outbreaks could be explained by the transient character of the observed stimulation. Liman and Rautenschlein [7] analyzed turkeys from a breeder flock immunized against TRT, and vaccinated the birds at the age of 33 days when specific anti-aMPV IgY antibodies could still be detected in their serum. Their findings corroborate our observations and indicate that the presence of maternal antibodies somehow protects the upper respiratory tract of turkeys against infiltration by CD8^+^ T cells regardless of the birds’ age. In this context, the presence of specific antibodies could contribute to the induction of aMPV/A immunophagocytosis through opsonization of the vaccine virus. The above observation is additionally validated by faster viral clearance in the upper respiratory tract and a significant decrease in MDA levels in MDA+0/V birds after vaccination.

Cha [17] demonstrated that inoculation of two-week old MDA- turkeys with aMPV/C increased the percentage of CD8^+^ T cells in nasal turbinate mucosa, without inducing any changes in the percentage of CD4^+^ T cells. Additionally, an increase in absolute counts of CD4^+^ and CD8^+^ T cells was reported in HG in MDA- chickens inoculated with aMPV/A or B [9], which is consistent with our results in MDA- vaccinated groups. The observed increase in absolute counts of both T cell subpopulations in HG and CD8^+^ T lymphocytes in TC could indicate that CD4^+^ T cells alone are unable to control the vaccine virus replication. Greater stimulation of vaccine-induced humoral immunity in MDA- vaccinated birds could indicate that the CD4^+^ T cells are unable to maintain such metabolic pressure, which triggers the additional CD8^+^ T cells infiltration in the upper respiratory tract of vaccinated turkeys.

Studies of other respiratory infections demonstrated that CD8^+^ T memory cells confer immunity to reinfection by restricting the spread of the virus at the site of its replication [22,23] but in turn, they may be directly responsible for damage to the host’s anatomical structures (e.g. mucous membranes) [24]. The question that arises in connection with our results is whether the presence of maternal antibodies interferes with the acquisition of T memory cells after vaccination against TRT. Further research is needed to examine the role of CD4^+^ and/or CD8^+^ T memory cells in protection against aMPV infections.

As demonstrated earlier, the TRT virus has immunosuppressive activity [25-28]. In our study a significant decrease in CD4^+^ and/or CD8^+^ T ACC in tracheal mucosa was noted in vaccinated birds 3–7 DPV in Experiments II - IV. Although, we can not assume that the above changes are indicative of T cell immunosuppression we may speculate that attenuated aMPV strains could also deliver such effects. Further research is necessary to establish whether aMPV vaccine strains possess immunosuppressive activity and if this activity *in vivo* could depend on specific antibody levels and birds’ age on the day of vaccination.

The role of double positive CD4^+^CD8^+^ T cells in the host’s immune response has not yet been fully elucidated, but in some production lines of hens those cells were found to represent a significant percentage of T lymphocytes in secondary immune structures [29]. Double-positive T cells could also have regulatory functions or could play the role of both T helper cells and cytotoxic T cells [30]. As it turned out in our study, this parameter varied significantly only in MDA- vaccinated turkeys.

Although most of the cited authors concluded their studies based on relative T cell count and not ACC, these values are highly proportional. Therefore it can be concluded that differences exist in the development and activation of CMI in the upper respiratory tract of turkeys vaccinated against TRT, and that those variations are correlated mainly with the birds’ age and maternal antibody level on the day of vaccination. The cited experiments produced similar results despite the use of different aMPV strains, which seems to suggest that differences in TRT immunopathogenesis are not strain-related [6,7,17].

In our study, MDA+0/V was the only group of vaccinated birds that did not produce anti-aMPV IgY antibodies in response to vaccination. On day 3 PV, anti-aMPV antibody level in the serum of MDA+0/V birds was significantly lower in comparison with unvaccinated birds. Those findings are partly consistent with results reported by Cook et al. [12] who did not observe an increase in serum levels of specific anti-aMPV antibodies in most MDA+ chickens vaccinated against TRT on the first day of life. In our experiment, a faster decrease in MDA levels in MDA+0/V birds after vaccination could be attributed to immunophagocytosis.

MDA+14/V and both MDA- vaccinated groups of turkeys responded to vaccination by producing specific IgY anti-aMPV antibodies that were detected in the serum (but not in tracheal washings) at 100-fold dilution on 14 DPV. In MDA-14/V birds, the level of those antibodies were elevated already on day 7 PV. Those results indicate that the level and the time required to induce humoral immunity after TRT vaccination are determined by age and MDA level on the day of vaccination. The above could impair early assessment of vaccine-induced immunity against TRT based on the results of routine serological monitoring which also has other disadvantages [4,31-33].

The absolute count of IgM^+^ B cells is a general, non-specific indicator of humoral immunity stimulation after vaccination. In this study, the only group of birds in which this parameter did not change or decreased significantly was MDA+0/V. A significant decrease in IgM^+^ B ACC in HG was also reported in the MDA-14/V group on day 14 PV, but in this case, it was probably compensated by intensive IgM^+^ B cell infiltration in tracheal mucosa.

Secretory IgA plays the main role in humoral immunity of upper respiratory tract. IgA^+^ B cells and specific IgA participates in the local immune response against TRT in the upper respiratory tract [15]. Absolute counts of IgA^+^ B cells and the levels of specific IgA were not determined in this study, but it could be hypothesized that, similarly to IgM^+^ B cell infiltration and specific IgY production, the production of specific IgA could also depend on the MDA level and the age of birds on the day of vaccination against TRT. Further research is needed to determine the involvement of IgA^+^ B cells and the role of IgA in protection against aMPV infections.

The observed differences in aMPV/A replication in nasal choanae between the MDA+0/V and MDA-0/V groups could be associated with high antibody levels in MDA+ birds upon vaccination. High levels of the these immunoglobulins probably facilitated and stimulated (upon immunophagocytosis) viral clearance from the upper respiratory tract, as suggested earlier by Cook et al. [11,12].

In both groups vaccinated on 14 DOL, aMPV/A RNA was practically undetectable in choanal swabs. Despite the above, the upper respiratory tract immune system was strongly stimulated in those groups after vaccination. Those results partially corroborate the findings of Rubbenstroth and Rautenschlein [16] who reported very high levels of vaccine-induced immunity against aMPV infections despite the fact the aMPV/A RNA was not regularly detected in nasal turbinates of turkeys vaccinated against TRT on 13 or 14 DOL. Differences between birds vaccinated at different age suggest that the replication of vaccine aMPV/A is limited during the maturation of the immune and/or respiratory system, but this does not influence the development of vaccine-induced immunity.

This work has some limitations. Given the fact that MDA+ and MDA- turkeys had different origin we were unable to perform statistical analysis of assayed parameters between them. However we could trace the diversity of their development after vaccination in each experiment in comparison to adequate not vaccinated control group. To ensure the reproducibility of the results, in each experiment, before the vaccination the birds were confirmed to be free (with PCR technique) from other common respiratory pathogens *Mycoplasma spp., Ornithobacterium rhinotracheale, Bordetella avium* and Newcastle Disease Virus (data not shown). Experiments I and III as well as II and IV were performed simultaneously. Additionally, birds for experiment I and II as well as III and IV were obtained from the hatcheries at the same time.

## Conclusion

Previous studies demonstrated that, under laboratory conditions, the live attenuated vaccines are highly efficient in protecting against TRT [7,9,17,21]. On the other hand, despite vaccination, TRT outbreaks are frequently observed in the field [7-9], which may stem not only from aMPV high virulence but, additionally, from differences in post-vaccination immunity development in birds with diversified immunological status and from the impact of other negative environmental and microbiological agents.

From the perspective of our study, at least a few pathomechanisms of this adverse situation may be concluded: (I) possible immunosuppressive activity of aMPV vaccine strains causing disorders in T cells activity or T cells activation, (II) possible disorders in antigen specificity acquisition of B and/or T lymphocytes in MDA+ groups, (III) high infiltration of CD8^+^ T lymphocytes in TC of MDA- groups, that (most likely) is not neutral to the morphology of tracheal mucosa, as well as (IV) temporary character of CD4^+^ T lymphocytes increase in the upper respiratory tract of MDA+ birds.

## Abbreviations

ACC: Absolute cell count
ALC: Absolute lymphocyte count
aMPV: Avian Metapneumovirus
CMI: Cell mediated immunity
DPV: Days post vaccination
FBS: Fetal bovine serum
HG: Harderian gland
IFN: Interferon
IL: Interleukin
MDA: Maternally derived antibodies
PBS: Phosphate-buffered saline
PV: Post vaccination
RCC: Relative cell count
RT-PCR: Reverse transcriptase polymerase chain reaction
(S/P)-ratio: Sample to positive ratio
TC: Tracheal mucosa
TCID: Tissue culture infectious dose
TRT: Turkey rhinotracheitis

## References

[1] Pedersen JC, Reynolds DL, Ali A (2000). The sensitivity and specificity of a reverse transcriptasepolymerase chain reaction assay for the avian pneumovirus (Colorado strain). Avian Dis.

[2] Buys SB, Du Preez JH (1980). A preliminary report on the isolation of a virus causing sinusitis in turkeys in South Africa and attempts to attenuate the virus. Turkeys.

[3] Collins MS, Gough RE, Alexander DJ (1993). Antigenic differentiation of avian pneumovirus isolates using polyclonal antibody and mouse monoclonal antibodies. Avian Pathol.

[4] Cook JKA, Jones BV, Ellis MM, Li J, Cavanagh D (1993). Antigenic differentiation of strains of Turkey rhinotracheitis virus using monoclonal antibodies. Avian Pathol.

[5] Seal BS (1998). Matrix protein gene nucleotide and predicted amino acid sequence demonstrate that the first US avian pneumovirus isolate is distinct from European strains. Virus Res.

[6] Smialek M, Tykalowski B, Stenzel T, Koncicki A (2012). The perspective of immunoprophylaxis and selected immunological issues in the course of the turkey rhinotracheitis. Pol J Vet Sci.

[7] Liman M, Rautenschlein S (2007). Induction of local and systemic immune reactions following infection of turkeys with avian Metapneumovirus (aMPV) subtypes A and B. Vet Immunol Immunopathol.

[8] Catelli E, Lupini C, Cecchinato M, Ricchizzi E, Brown P, Naylor CJ (2010). Field avian metapneumovirus evolution avoiding vaccine induced immunity. Vaccine.

[9] Rautenschlein S, Aung YH, Haase C (2011). Local and systemic immune responses following infection of broiler-type chickens with avian Metapneumovirus subtypes A and B. Vet Immunol Immunopathol.

[10] Jones RC, Wolliams RA, Baxter-Jones C, Savage CE, Wilding GP (1988). Experimental infection of laying turkeys with rhinotracheitis virus: distribution of virus in the tissues and serological response. Avian Pathol.

[11] Cook JKA, Ellis MM, Dolby CA, Holmes HC, Finney PM, Huggins MB (1989). A live attenuated turkey rhinotracheitis virus vaccine. 1.Stability of the attenuated strain. Avian Pathol.

[12] Cook JKA, Holmes HC, Finney PM, Dolby CA, Ellis MM, Huggins MB (1989). A live attenuated turkey rhinotracheitis virus vaccine. 2. The use of the attenuated strain as an experimental vaccine. Avian Pathol.

[13] Jirjis FF, Noll SL, Halvorson DA, Nagaraja KV, Shaw DP (2002). Pathogenesis of avian pneumovirus infection in turkeys. Vet Pathol.

[14] Jones RC, Naylor CJ, al-Afaleq A, Worthington KJ, Jones R (1992). Effect of cyclophosphamide immunosuppression on the immunity of turkeys to viral rhinotracheitis. Res Vet Sci.

[15] Cha RM, Khatri M, Sharma JM (2007). B-cell infiltration in the respiratory mucosa of turkeys exposed to subtype C avian metapneumovirus. Avian Dis.

[16] Rubbenstroth D, Rautenschlein S (2010). Compromised T-cell immunity in turkeys may lead to an unpredictable avian metapneumovirus vaccine response and variable protection against challenge. Avian Pathol.

[17] Cha RM. Immunopathogenesis of avian Metapnumovirus in the turkeys. PhD thesis. University of Minnesota, United States; 2009.

[18] Cavanagh D, Mawditt K, Britton P, Naylor CJ (1999). Longitudinal field studies of infectious bronchitis virus and avian pneumovirus in broilers using type-specific polymerase chain reactions. Avian Pathol.

[19] Gough RE, Jones RC, Saif YM (2008). Avian Metapneumovirus. Diseases of poultry.

[20] Wiliams RA, Savage CE, Jones RC (1991). Development of a live attenuated vaccine against turkey rhinotracheitis. Avian Pathol.

[21] Patnayak DP, Sheikh AM, Gulati BR, Goyal SM (2002). Experimental and field evaluation of a live vaccine against avian pneumovirus. Avian Pathol.

[22] Kimpen JL, Rich GA, Mohar CK, Ogra PL (1992). Mucosal T cell distribution during infection with respiratory syncytial virus. J Med Virol.

[23] de Bree GJ, van Leeuwen EM, Out TA, Jansen HM, Jonkers E, van Lier RA (2005). Selective accumulation of differentiated CD8+ T cells specific for respiratory viruses in the human lung. J Exp Med.

[24] Scott KG, Buret AG (2004). Role of CD8+ and CD4+ T lymphocytes in jejunal mucosal injury during murine giardiasis. Infect Immun.

[25] Timms LM, Jahans KL, Marshall RN (1986). Evidence of immunosuppression in turkey poults affected by rhinotracheitis. Vet Rec.

[26] Chary P, Rautenschlein S, Njenga MK, Sharma JM (2002). Pathogenic and immunosuppressive effects of avian pneumovirus in turkeys. Avian Dis.

[27] Chary P, Rautenschlein S, Sharma JM (2002). Reduced efficacy of hemorrhagic enteritis virus vaccine in turkeys exposed to avian pneumovirus. Avian Dis.

[28] Marien M, Decostere A, Martel A, Chiers K, Froyman R, Nauwynck H (2005). Synergy between avian pneumovirus and Ornithobacterium rhinotracheale in turkeys. Avian Pathol.

[29] Davidson F, Kaspers B, Schat KA (2008). Avian immunology.

[30] Parel Y, Chizzolini C (2004). CD4+ CD8+ double positive (DP) T cells in health and disease. Autoimmune Rev.

[31] Eterradossi N, Toquin D, Guittet M, Bennejean G (1995). Evaluation of different turkey rhinotracheitis viruses used as antigens for serological testing following live vaccination and challenge. Zentralbl Veterinarmed B.

[32] Mekkes DR, de Wit JJ (1998). Comparison of three commercial ELISA kits for the detection of turkey rhinotracheitis virus antibodies. Avian Pathol.

[33] Cook JKA (2000). Avian rhinotracheitis. Rev Sci Tech.

